# Research on robust fault-tolerant control of the controllable suspension based on knowledge-data fusion driven

**DOI:** 10.1038/s41598-023-50082-8

**Published:** 2023-12-20

**Authors:** Honglin Zhu, Weiping Ding, Mingliang Yang, Yudong Wu, Tong Du

**Affiliations:** 1 School of Mechanical Engineering, Southwest Jiaotong University, Chengdu, 610031 Sichuan China; 2 National Laboratory for Rail Transportation, Southwest Jiaotong University, Chengdu, 610031 Sichuan China

**Keywords:** Electrical and electronic engineering, Mechanical engineering

## Abstract

For the robust fault-tolerant control of the controllable suspension system, a control strategy driven by knowledge-data fusion is proposed. Firstly, the boundary fuzziness between perturbation type uncertainty and gain type fault is analyzed, and then a data-driven method is introduced to avoid the state estimation of system uncertainty and fault. The proximal policy optimization algorithm in reinforcement learning is selected to construct a “data control law”, to deal with uncertainty and fault. On the other hand, based on the classical sky-hook control, the “knowledge control law” for system performance optimization is designed, taking into account the nonlinear and non-stationary characteristics of the system. Furthermore, the dependency between robust fault tolerance and performance optimization control is revealed, and the two control laws are fused by numerical multiplication, to realize the performance matching optimization control of robust fault tolerance of controllable suspension system driven by knowledge-data fusion. Finally, the effectiveness and feasibility of the proposed method are verified by the simulation and real-time experiment of non-stationary excitation and near-stationary excitation under the combination of uncertainty and fault.

## Introduction

The controllable suspension system is an effective technical method to improve vehicle ride comfort and stability^1^, has attracted much attention in academia and industry, and relevant researches cover performance optimization control^2,3^ and robust fault-tolerant control. With the development and application of controllable suspension systems, academia and industry focus more on the research of robust fault-tolerant control methods. Among them^4–20^, robustness is oriented to the uncertainty factors such as perturbation, time delay, and external interference of the system, which interaction and superposition will directly lead to performance degradation. Fault-tolerant is for the system fault, it is divided into gain type, bias type, and stuck type. The fault is related to uncertain factors, and the accumulation of uncertainty over time will increase the probability of fault, which will lead to performance deterioration or failure.

The suspension robust fault-tolerant control can be divided into two categories: First, “knowledge-driven control” based on mechanism model, empirical rules, and domain knowledge, including H∞ control based on state feedback or output feedback^6–8^, and H2/H∞ control^9,10^, fault-tolerant control based on state observer for estimation and fault reconstruction^11,12^, quantized non-fragile feedback control based on dynamic quantizer to deal with faults with or without actuators^13^, adaptive backstep control for the random fault of actuator^14^, and the sky-hook control, acceleration control, power drive control, and hybrid control with a certain passive fault tolerance ability^15–19^, a L-function is presented to avoid an effect of model uncertainties and the external disturbances^20^, etc. These control methods have perfect theoretical support, strong interpretability, and high execution efficiency, and are the beginning of the research on controllable suspension systems. However, they are mostly based on idealized uncertainty and fault hypothesis, which make the design of the relevant control law conservative and fail to fully utilize the performance of the controllable suspension system. Second, “data-driven control” based on neural networks, deep learning, and reinforcement learning, including the estimation and control of uncertainty by neural networks (NN) or adaptive neural networks^21–25^, backstep control law based on radial basis function (RBF) neural network mini learning^26^, model-free robust control combining NN neural network and Q-like learning^27^, intelligent control based on improved Extreme Learning Machine (ELM) to approximate and compensate nonlinear unmodeled dynamics^28^, etc. These control methods do not require accurate modeling, have strong universality, and are good at dealing with black or grey box problems, but they have poor interpretability due to the difficulty of theoretical analysis, and they have a strong dependence on the quantity and quality of training data, weak generalization, and time-consuming training.

Both knowledge-driven and data-driven control methods have advantages and disadvantages. Both control methods use state estimation when dealing with uncertainty and fault, and the premise of real-time fault diagnosis and recognition should be based on the cognition of the relationship between uncertainty and fault. However, both uncertainty and fault are time-varying, and the mathematical description methods of perturbation type uncertainty and gain type fault are similar, with overlapping areas, which are not easy to diagnose and identify in a deterministic way and can be dealt with by fuzzy means. Therefore, the data-driven estimation method of uncertainty processing in the above literature is desirable^21–25^, but it has not effectively solved the problem of distinguishing uncertainty and fault. In addition, for the application of the data-driven method, it is necessary to combine the mechanism of the controlled object. For the grey box or black box problem whose mechanism is fuzzy and difficult to clarify, the advantage of data-driven is prominent. For the white box problem with a clear mechanism, we should follow the prior experience and use knowledge drive to control it.

The problem of robust fault-tolerant control of the controllable suspension system includes both white box parts with clear mechanisms and black box parts with fuzzy mechanisms. Therefore, based on the principle of “white box problem be controlled by knowledge-driven, black box problem controlled by data-driven”, the robust fault-tolerant control law of controllable suspension is divided into two parts: “knowledge control law” and “data control law”. In other words, based on the dynamic characteristics of the controllable suspension system, the knowledge control law is designed with empirical rules, which is fully conducive to strong theoretical analysis support, ensuring the explainability of the control law, making the control strategy “transparent”, and ensuring the trust in the use of the strategy. For the ambiguity of the correlation and distinction between uncertainty and fault, and the unclear mechanism, it is classified as a kind of black box problem, and based on sorting out its qualitative correlation, a data-driven data control law is designed to establish a direct correlation between information input containing uncertainty and control output, eliminating the process of estimating and identifying uncertainty and fault. The control without an estimator is realized, the control loop is simplified, and the operation efficiency is improved. Then, the fusion of the two parts is used as the final robust fault-tolerant control law, forming a new knowledge-data fusion driven architecture.

The main contributions are as follows: 1) Distinct from the existing studies, based on the boundary fuzziness analysis of perturbation type uncertainty and gain type fault of the controllable suspension system, a data-driven method is introduced to avoid the state estimation of system uncertainty and fault, and a robust fault-tolerant data control law is constructed by it. Furthermore, in order to improve the inexplicability of data-driven, a knowledge control law for system performance optimization is designed based on the knowledge-driven method, taking into account the nonlinearity and non-stationary characteristics of the system. Then, based on the multiplicity characteristic of uncertainty and fault in the damping force characterization formula of the adjustable shock absorber, the two parts are multiplied to complete the fusion of the control law. 2) Based on the order relation method and empirical preference construct the fusion reward function under the knowledge-data-driven architecture, involving the suspension performance indicators: body vertical acceleration, suspension dynamic travel displacement, and wheel acceleration. At the same time, the feedback effect of robust fault-tolerant control law is taken into account based on the hard constraint of the adjustable current boundary of the damper in the controllable suspension system. 3) Based on the weight coefficients among the three key performance indicators of suspension, a weighted normalized index is constructed, and then the normalized indicators under the combination of uncertainty and fault are linearly superimposed to obtain the arithmetic average, which is used as an effective evaluation index of the robust fault-tolerant of the proposed knowledge-data driven control strategy. The effectiveness and feasibility of the proposed method is verified through the simulation and real-time experiment analysis of knowledge-data fusion driven robust fault-tolerant control, knowledge-driven control, and data-driven control under different excitation road surfaces, different uncertainty and fault combinations.

The subsequent contents of this paper are organized as follows: Section “Uncertainty and fault analysis of the controllable suspension system” analyzes the uncertainty and fault characteristics of the controllable suspension system; Section “Robust fault-tolerant control architecture and strategy” designs a knowledge-data driven robust fault-tolerant control architecture and control strategy; Section “Demonstrative example” builds a nonlinear, non-stationary, and uncertain interactive simulation and real-time experiment environment of the controllable suspension system to verify the effectiveness and feasibility of the proposed control strategy; Section “Conclusion” summarizes the research content.

## Uncertainty and fault analysis of the controllable suspension system

**Figure 1:**
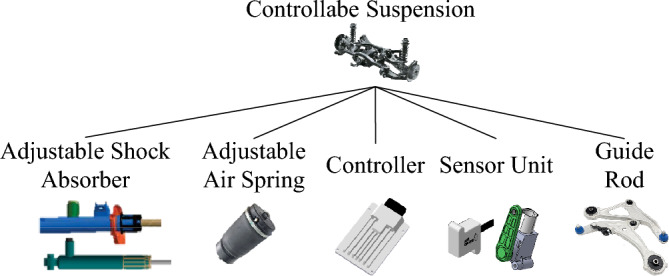
Controllable suspension system composition.

The composition of the controllable suspension system is shown in Fig. 1, which generally includes the sensor unit, controller, actuator (adjustable air spring, adjustable shock absorber) and guide rod, etc. According to whether the actuator can actively generate force, it can be divided into active suspension or semi-active suspension, wherein semi-active suspension can be divided into adjustable damping, adjustable stiffness, adjustable height, adjustable damping, and stiffness, and height according to controllable parameters. In this paper, the adjustable damping suspension system is taken as the object of relevant research. The difference between it and the traditional passive suspension, the first is that its damping force is determined by the speed of the shock absorber and the control current, which results in the superposition of two kinds of nonlinear characteristics. The second is the introduction of the electronic control unit, which increases the probability of uncertainty and fault. The interaction of the above two points increases the challenge of robust fault-tolerant control.

### Nonlinear characteristics of the controllable suspension system

**Figure 2:**
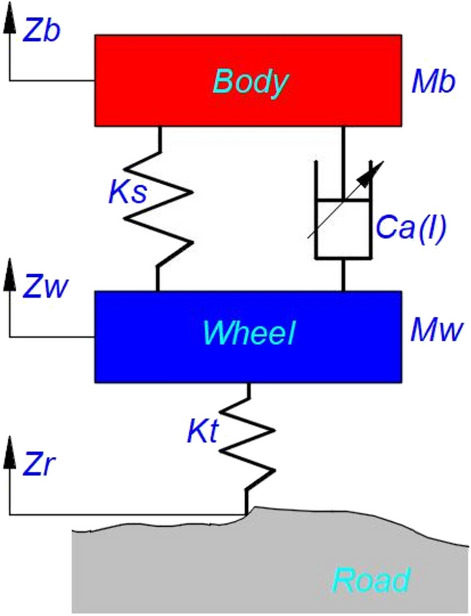
Two-degree-of-freedom nonlinear controllable suspension model.

The controllable suspension system is a complex system with nonlinear, time-varying, and uncertain characteristics, and the more comprehensive system characteristics are considered, the more closer to the real suspension state, but the more difficult to analyze and solve. The more simplified the model, the easier to analyze and solve, but the accuracy of the system will be reduced. Therefore, according to the analysis requirement of this subject, which focuses on vertical control of damping adjustable suspension, referring to existing research^15,17,18,28,29,32^, the modeling principle is defined as follows: (1) Suspension bushing stiffness is not considered. (2) The wheel damping is not considered, and its stiffness is defined as linear stiffness. (3) Non-linearity due to the installation Angle of key suspension components is not considered. Therefore, the two-degree-of-freedom suspension model shown in Fig. 2 is taken as the basic model, as shown in Eq. (1).

1 $$\left\{ \begin{gathered} M_{b} \ddot{z}_{b} = - K_{s} \left( {z_{b} - z_{w} } \right) - C_{a} \left( I \right)\left( {\dot{z}_{b} - \dot{z}_{w} } \right) \hfill \\ M_{w} \ddot{z}_{w} = K_{s} \left( {z_{b} - z_{w} } \right) + C_{a} \left( I \right)\left( {\dot{z}_{b} - \dot{z}_{w} } \right) - K_{t} \left( {z_{w} - z_{r} } \right) \hfill \\ \end{gathered} \right.$$

where *M*_*b*_ is the equivalent body mass, also the sprung mass, the unit is kg. *M*_*w*_ is the equivalent wheel mass or unsprung mass, the unit is kg. *z*_*b*_、*z*_*w*_ and *z*_*r*_ are the vertical displacements of the body, wheels, and Road, the unit is mm. *K*_*s*_ is the suspension stiffness unit N/mm. When the suspension stiffness *K*_*s*_ is linear, its value is constant and does not change with the displacement of the suspension wheel jump. When the nonlinear value of *K*_*s*_ is taken, its value can be expressed as a function related to displacement, which changes with the travel displacement of the suspension. In the linear segment, its linear value is the same as that of linear *K*_*s*_. The difference is reflected in the position near the jump up and down limit, and its stiffness value will increase rapidly. Whether *K*_*s*_ is a linear or nonlinear value, it needs to rely on the analysis condition. If the analysis condition is a flat road with constant speed, the suspension travel displacement is generally within ± 10 mm and has been in the linear section, it is not necessary to define *K*_*s*_ as a nonlinear value. However, when the vehicle is driving under non-stationary large excitation, such as repairing asphalt road, manhole cover road, joint road, pothole road, and speed bump, the suspension travel is large, generally exceeding ± 30 mm, so that it enters the nonlinear section of suspension stiffness, *K*_*s*_ is required to define as the nonlinear value. *C*_*a*_(*I*) is the damping coefficient of the adjustable damping suspension system, which is a function of current, and its value changes with the change of current to achieve timely adjustable damping, the unit is N·s/m. It also can be expressed as "linear" controllable and “nonlinear” controllable. For *C*_*a*_(*I*), whether the linear value or nonlinear value is adopted, also depends on the analysis condition. If the analysis condition is the flat road with constant velocity, the shock absorber velocity is generally less than ± 0.10 m/s, under the valve opening velocity point of the shock absorber (generally 0.10–0.20 m/s), then the damping coefficient before the valve opening point of the shock absorber can be used as a linear damping coefficient, and *C*_*a*_(*I*) need not be defined as a nonlinear value. However, when the vehicle is running under non-stationary large excitation such as repairing asphalt road, manhole cover road, joint road, pothole road, and speed bump, the shock absorber velocity is relatively large, generally exceeding ± 0.20 m/s, so that it works after the valve opening velocity of the suspension shock absorber. If the linear damping average damping coefficient is assigned, it is insufficient to characterize the damping force value of the shock absorber. Especially in the medium and low-velocity segment (0 ~  ± 0.60 m/s), *C*_*a*_(*I*) is required to define as a nonlinear value.

**Figure 3:**
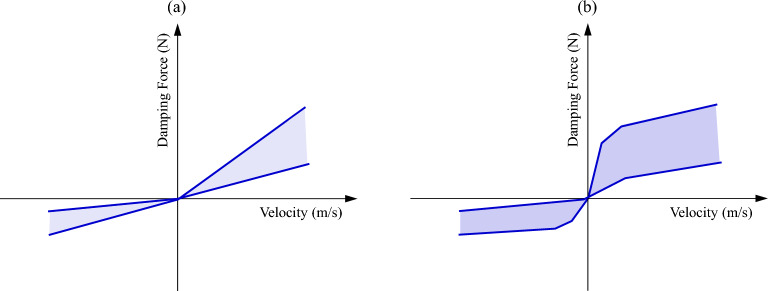
The adjustable damping: (**a**) linear, and (**b**) nonlinear.

As shown in Fig. 3, when the adjustable damping *C*_*a*_(*I*) takes a linear value, the damping coefficients of both the recovery section and the compression section are an interval value, which generally increases with the increase of the current. Although the damping value is linear, the introduction of the current dimension makes it nonlinear (current adjustable nonlinear). Further, when the damping coefficients *C*_*a*_(*I*) take a nonlinear value, the nonlinear brought by the velocity dimension and the current dimension are superimposed to form a “variable” nonlinear or “interval” nonlinear, *F*(*I,v*) relationship (the nonlinear relationship between damping force and current and velocity), which enhances the nonlinear characteristics of the suspension. It is different from the damping nonlinear *F*(*v*) relation defined in the conventional system of passive damping (nonlinear relation of damping force *vs.* velocity).

**Figure 4:**
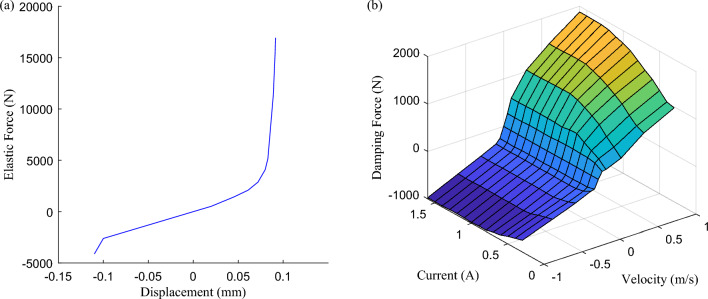
The nonlinear parameters of suspension: (**a**) nonlinear suspension stiffness *K*_*s*_, and (**b**) nonlinear suspension damping *Ca*(*I*).

According to the above analysis, relevant research on adjustable damping suspension is carried out for non-stationary excitation. Both suspension stiffness and suspension damping should be assigned nonlinear values, as shown in Fig. 4, and relevant vehicle parameters are shown in Table 1.

**Table 1:** Vehicle parameter list.

Vehicle parameter	Nominal value	Unit
Sprung mass* M*_*b*_	423.5	kg
Unsprung mass *M*_*w*_	56.0	kg
Nonlinear suspension stiffness *K*_*s*_	Figure 4a	–
Linear tire stiffness *K*_*t*_	225,000.0	N/m
Nonlinear suspension damping *CA*(*I*)	Figure 4b	–

**Figure 5:**
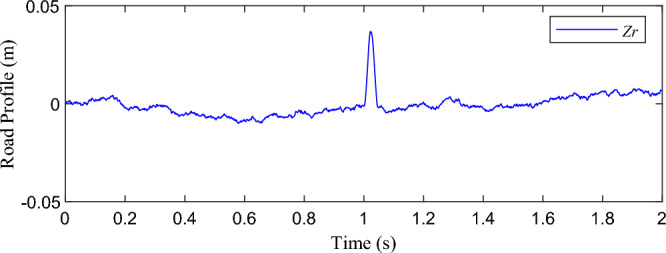
Time domain model of B-class pavement and speed bump.

The non-stationary excitation conditions were constructed with the B-class pavement and speed bump composite time-domain model, as shown in Fig. 5. Equation (2) is the time domain model of speed bump, and Eq. (3) is the time domain model of B-class pavement, *h*_*bump*_ is the speed bump section height, unit is m, generally be 0.04 m. *l*_*bump*_ refers to the width of the speed bump, the unit is m, generally be 0.40 m. *V*_*el*_ is the velocity of the vehicle, unit m/s. *t* is the time, the unit is s, *z*_*r*_(*t*) is the single-wheel road excitation, and the unit is m. *f*_*min*_ = *n*_*l*_*V*_*el*_, *n*_*l*_ is the lower limit of pavement space cutoff frequency, which is 0.011 m^-1^. *w*(*t*) is white noise. *n*_*0*_ is the reference spatial frequency (0.1 m^-1^). *G*_*q*_(*n*_*0*_) is the road roughness coefficient, the unit is m^3^.

2 $$h\left( t \right) = \left\{ \begin{gathered} 0\quad \quad \quad \quad \quad \quad \quad \quad \quad \quad \quad \;t \le 0~\;and\;t \ge 1 + l_{{bump}} /V_{{el}} \hfill \\ \frac{{h_{{bump}} }}{2}\left( {1 - cos\frac{{2\pi v}}{{l_{{bump}} }}t} \right)\quad \;~1 \le t \le 1 + l_{{bump}} /V_{{el}} \hfill \\ \end{gathered} \right.$$

3 $$\dot{{z}_{r}}\left(t\right)=-2\pi {f}_{min}{z}_{r}\left(t\right)+2\pi {n}_{0}\sqrt{{G}_{q}\left({n}_{0}\right){V}_{el}} w(t)$$

**Figure 6:**
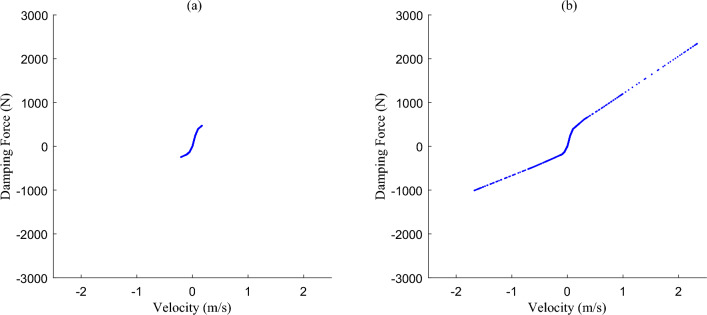
The damping force distribution of nonlinear nominal model simulation results: (**a**) B-class pavement excitation segment—nearly linear, and (**b**) Speed bump excitation segment—nonlinear.

**Figure 7:**
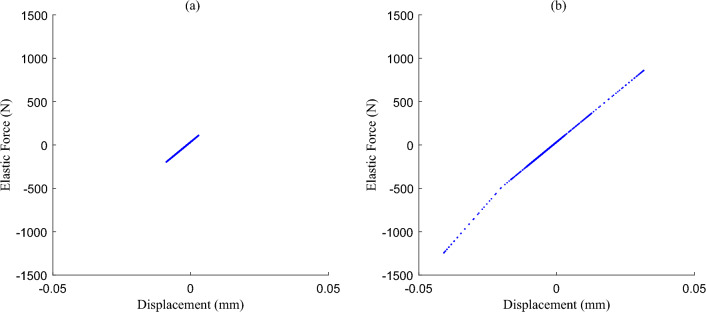
The elastic force distribution of nonlinear nominal model simulation results: (**a**) B-class pavement excitation segment-nearly linear, and (**b**) Speed bump excitation segment-nonlinear.

Based on the excitation of non-stationary excitation conditions constructed, the simulation results of the damping force and elastic force simulated by the nonlinear nominal model are shown in Figs. 6 and 7. According to the excitation input in Fig. 5, in the excitation section of B-class pavement, the shock absorber velocity is about ± 0.1 m/s, the dynamic travel displacement of the suspension is about ± 10 mm, and the force values are in the near-linear section. When the absolute value of shock absorber velocity exceeds 1 m/s, the absolute value of suspension dynamic travel displacement exceeds 30 mm, and the force values enter the nonlinear section. In accordance with the above analysis, it is further explained that the nonlinear characteristics of the suspension system should be considered when non-stationary excitation conditions with a large impact.

### Uncertainty and fault boundary fuzziness of the controllable suspension system

The uncertainty and fault of the controllable suspension system exist in every process of the control system, from input acquisition, and control operation to output execution. Uncertainty is usually divided into time-delay type and perturbation type. The time-delay type includes the sampling time of the sensor unit, control operation time, control current response time of the solenoid valve, and damping force response time of the shock absorber. The perturbation type uncertainty includes signal perturbation of the sensing unit, the value perturbation of the driving current, and absorber damping force. The fault is divided into gain type, bias type, and stuck type. As shown in Table 2, the gain fault is manifested by scaling of the sensor unit signal, the value of solenoid valve driving current, and the shock absorber damping force, the offset fault is manifested by the deviation of the above-related values from the normal dynamic balance position, and the stuck fault is manifested by the output of the related values at a constant value.

**Table 2:** Uncertainty and fault table.

No.	Uncertainty/fault		Remarks
1	Time-delay type	$x(t+\tau \left(t\right))$	Uncertainty: sensor unit/control operation/current response/damping force response
2	Perturbation type	$x\times (1+\delta (t))$	Uncertainty: sensor unit /current/damping force
3	Gain type	$x\times \sigma$	Fault: sensor unit/current/damping force
4	Bias type	$x+\rho$	
5	Stuck type	$\rho$	

*In the table, $\tau$ is the delay, $\delta$ is the perturbation, $\sigma$ is the fault constant gain, $\rho$ is the fault constant bias.

When the uncertainty and fault are transmitted to the output execution along each process of the control system, both intensification and attenuation exist. For example, intensification is reflected in the cumulative superposition of time-delay type uncertainty along the path transmission process from sensor acquisition and control operation to output execution, and the final phenomenon is that the time delay of output execution increase. Attenuation is reflected in the output of adjustable shock absorber damping force, whose damping force value is affected by two perturbations, one is the perturbation of the value of the control current, and the other is the perturbation of the damping force value caused by the design and manufacturing error of the shock absorber. In the superposition fusion process of these two parts, the influence of the control current perturbation will be weakened. Therefore, the uncertainty and fault analysis focuses on the actuator unit of the adjustable damping suspension system, the solenoid valve type adjustable shock absorber, and takes it as the research object.

**Figure 8:**
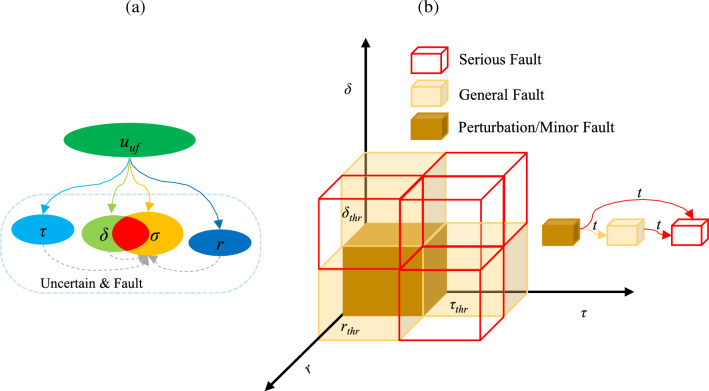
The relationship: (**a**) the output damping force with uncertainty and fault, and (**b**) uncertainty and fault sets.

On the one hand, the output damping force (*u*_*uf*_) of the solenoid-valve type adjustable shock absorber is determined by the external input excitation (*r*), internal uncertainty, and fault, as shown in Fig. 8a. Clarify the relationship between uncertainty and fault: First, the perturbation type uncertainty is very similar to the gain fault (Table 2) in the mathematical description. In general, $\delta$ represents the perturbation of the damping force value caused by the manufacturing error or the change in the ambient temperature of the shock absorber, $\delta \in [-\mathrm{0.2,0.2}]$. $\sigma$ represents the fault caused by factors such as oil deterioration or oil leakage of the shock absorber or solenoid valve spool displacement deviation, $\sigma \in [\mathrm{0,1}]$. The perturbation type uncertainty range overlaps with the gain type fault range, and is eventually input to the suspension system through damping force, which is difficult to distinguish.

Second, the occurrence of the fault will be affected by the time delay, perturbation, and external input excitation. It is assumed that the time delay and perturbation are mapped to the fault under the action of the external input excitation, as shown in Fig. 8b. *τ*, *δ* and *r* correspond to one-dimensional coordinate axes respectively, and the corresponding regions are divided into perturbation/minor fault, general fault and serious fault according to the critical values of each axis (*τ*_*thr*_, *δ*_*thr*_, *r*_*thr*_). Under the premise of a certain cumulative time history, the perturbation/minor fault overlap zone is defined as if the three-dimensional values are all within the critical value, and the general fault is defined as if and only if there is one dimension beyond the critical value, the serious fault is more than two dimensions beyond the critical value, and the state of the perturbation/minor fault, the general fault and the serious fault are gradually transferred over the time history. However, be same as the traditional method, there are still problems with how to demonstrate the rationality of the hypothesis of uncertainty and fault probabilities, and how to obtain the real-time value of the shock absorber damping force. For such “black box” problem, based on qualitative relationship analysis, we can set a new way to avoid online diagnosis and identification of uncertainty and fault. The idea, with the help of the data-driven method, establishes the correlation between the input signal and the control law. The uncertainty such as time delay *τ*, perturbation *δ,* and external excitation *r* are taken into account with the input signal, that is, the fault is fuzzy as a process quantity, without diagnosis and identification.

## Robust fault-tolerant control architecture and strategy

Based on the analysis of uncertainty and fault characteristics of the controllable suspension system in the previous section, a new robust fault-tolerant architecture and strategy is proposed. Based on the principle of “white box problem be controlled by knowledge-driven, black box problem controlled by data-driven”, the robust fault-tolerant control law of uncertain systems is divided into “knowledge control law” and “data control law”. The knowledge control law is designed with empirical rules to ensure the interpretability of the control law. The data control law is designed with the data-driven method, and the direct correlation between the input signal and control output containing uncertainty is established to avoid the uncertainty and fault state estimation of the system. It involves the delineation method and fusion mechanism of the two types of control laws, and the clarification of strategy realization.

### Knowledge-data fusion driven control for robust fault-tolerant architecture

Based on the boundary fuzziness analysis of perturbation type uncertainty and gain type fault of the controllable suspension system, a data-driven method is introduced to avoid the state estimation of system uncertainty and fault, and a robust fault-tolerant “data control law” is constructed. A “knowledge control law” for system performance optimization is designed based on the knowledge-driven method, taking into account the system’s nonlinearity and non-stationary characteristics. The fusion mechanism of the two control laws lies in the dependency between robust fault tolerance and performance optimization control, which can be started from the analysis of the damping force characteristics of the shock absorber in the adjustable damping suspension system. First, the nominal damping force ${u}_{d}$ of the adjustable shock absorber is defined, in Eq. (4).

4 $${u}_{d}=F\left(I(t),v(t)\right)$$

where *F*( ) is a function of the velocity and control current of the shock absorber as input, the unit is N. The symbol *I* is the control current, the unit is A. *v* is the velocity of the shock absorber, and the unit is m/s, determined by the external input excitation.

Further, on the basis of Eq. (4), ${u}_{ud}$ is the damping force of the shock absorber with uncertainty, including the time delay type and the perturbation type. The time delay type is inserted through the control current *I*. And the perturbation type is reflected in the final output damping force, see Eq. (5).

5 $${u}_{ud}=\left(1+\delta \left(t\right)\right)\cdot F\left(I(t+\tau (t)),v(t)\right)$$

At the same time, the fault expression in Table 2 is combined into the additive and multiplicative characteristic fault shown in Eq. (6). This expression defines the classification of the fault under the ideal assumed state, while the actual fault is time-varying, including the three ideal classification states, and the three are dynamically transformed. Here, it is more willing to unify them as the time-varying gain type $\sigma (t)$ except the stuck fault. Then the fault expression is converted into the multiplicative characteristic fault shown in Eq. (7), which can include the gain type and the bias type, and can also represent the dynamic change between them.

6 $${u}_{f}=\sigma \cdot {u}_{d}+\rho$$

7 $${u}_{f}=\sigma (t)\cdot {u}_{d}$$

By combining the Eqs. (5) and (7), the damping force expression of the shock absorber including uncertainty and fault can be obtained:

8 $${u}_{uf}=\sigma \left(t\right)\cdot \left(1+\delta \left(t\right)\right)\cdot F\left(I(t+\tau (t)),v(t)\right)$$

where ${u}_{uf}$ is the adjustable damping force of the shock absorber including uncertainty and fault, the unit is N.

**Figure 9:**
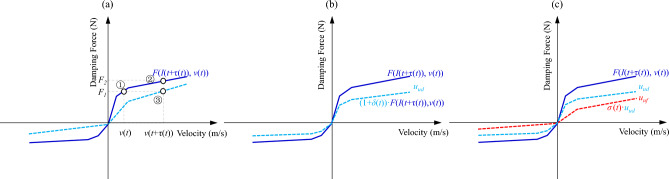
Adjustable damper force–velocity curve with uncertainty and fault: (**a**) time delay type uncertainty, (**b**) perturbation type uncertainty, and (**c**) gain type fault.

Based on Eq. (8), the influence relationship of uncertainty and fault on the damping force of the adjustable shock absorber can be obtained, as shown in Fig. 9.

Based on Fig. 9a, the influence of time-delay type uncertainty is analyzed, assuming that the expected damping force of the control system is ${F}_{1}$, when the control current $I(t)$ does not delay output of $\tau (t)$, the velocity of the shock absorber at time *t* is $v(t)$, corresponding to point ① in the figure, the damping force value is exactly ${F}_{1}$. However, there is a delay $\tau (t)$ in the actual system, then at the time $t+\tau (t)$, the shock absorber velocity is $v(t+\tau (t))$, corresponding to point ② in the figure, and the damping force is ${F}_{2}$, which deviates from the expectation. In order to make the damping force at $t+\tau (t)$ time output by ${F}_{1}$, at point ③ in the figure, then the damping force velocity curve $F\left(I(t+\tau (t)),v(t)\right)$ with the time delay can be scaled to output ${F}_{1}$, and the time delay type uncertainty can be compensated by means of product.

At the same time, from Fig. 9b,c, when perturbation type uncertainty or gain type fault occurs, it can be compensated by a certain proportion of scaled damping force. At all, the uncertainty and fault of adjustable damping suspension systems are uniformly described as “multiplicative characteristics”, which can be compensated by proportional multiplicataion based on performance optimization control. Specifically, the performance optimization output of the system is driven by knowledge, and the value of the control current *I* is determined. Compensating the influence of $\tau (t)$, $\delta \left(t\right)$, and $\sigma \left(t\right)$ with data driven, and the scaling ratio of the control current *I* is determined.

**Figure 10:**
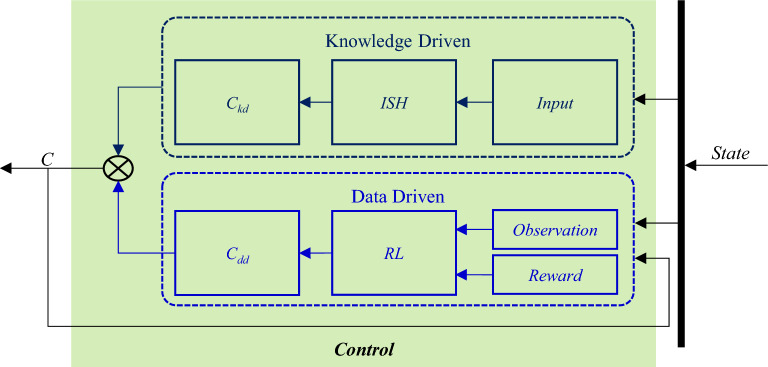
Robust fault-tolerant control architecture based on knowledge-data fusion driven.

Based on this, knowledge driven control and data driven control fusion through multiplication, the knowledge-data fusion driven robust fault-tolerant control architecture for the controllable suspension system is constructed, as shown in Fig. 10. This architecture is composed of two parts: knowledge-driven and data-driven, and the knowledge-driven control law *C*_*kd*_ (knowledge control law) is used to deal with suspension system performance optimization. The control law *C*_*dd*_ (data control law) is a data-driven control law that deals with uncertainty and fault in suspension control and deals with the black box problem of uncertainty and fault. The final control instruction *C* relies on the “multiplicative feature” of uncertainty and fault in Eq. (8), and multiplies *C*_*kd*_ and *C*_*dd*_ numerically. The “*state*” is the state variable of the controllable suspension system, and the “*control*” is a robust fault-tolerant controller.

The “*state*” includes body vertical acceleration ${\ddot{z}}_{b}$, suspension relative velocity ${\dot{z}}_{b}-{\dot{z}}_{w}$, suspension dynamic travel displacement ${z}_{b-}{z}_{w}$, etc. Knowledge driven consists of “*Input*” (input), “*ISH*” (modified sky-hook strategy), and *C*_*kd*_ (output). “*Input*” includes suspension relative velocity and body velocity from “*state*”, in which body velocity is obtained based on the sprung vertical acceleration of the body by Kalman filter^29^. Data-driven consists of “*Observation*”(observation input), “*Reward*”(reward and punishment rule), “*RL*”(Reinforcement Learning), and *C*_*dd*_ (output). The “*Observation*” includes relative velocity ${\dot{z}}_{b}-{\dot{z}}_{w}$, suspension dynamic travel displacement ${z}_{b-}{z}_{w}$, and robust fault-tolerant control law *C*, and the “*Observation*” includes time delay, perturbation, and external excitation uncertainty information, which be analyzed in the II*.B*. The “*Reward*” consists of two parts, one is based on the performance index of the controllable suspension system, and the other considers the feedback effect of the robust fault-tolerant control law *C*.

### Robust fault-tolerant control strategy driven by knowledge and data

Based on the knowledge-data fusion driven robust fault-tolerant control architecture of controllable suspension system, design a knowledge control law deal with performance optimization and a data control law deal with uncertainty and fault, to realize robust fault-tolerant control of controllable suspension system, and improve the comprehensive ride comfort of the vehicle under the influence of uncertainty and fault.

The main performance indexes of the suspension system include body vertical acceleration, suspension dynamic travel displacement, and wheel dynamic load. And the body vertical acceleration is an important basis for evaluating ride comfort and reducing its amplitude is conducive to improving vehicle comfort. The suspension dynamic travel displacement is related to its limit travel, and too large dynamic travel displacement will lead to the phenomenon of impacting the limit block, reducing the dynamic travel displacement and the frequency of impacting the limit block, which is conducive to improving the vehicle comfort. The dynamic load between the wheel and the road directly affects the adhesion effect between the wheel and the ground, which is related to the vehicle handling stability, reducing the dynamic load of the tire within a certain range is conducive to improving the vehicle handling stability.

The three performance indexes are all the smaller, the better, as shown in Eqs. (9), (1). where ${\ddot{z}}_{b}$ is the vertical acceleration of the body, ${z}_{b-}{z}_{w}$ is the dynamic travel displacement of the suspension, and ${\ddot{z}}_{w}$ is the acceleration of the wheel. Because it is not easy to obtain the ${z}_{w}-{z}_{r}$ of the dynamic load ${K}_{t}\left({z}_{w}-{z}_{r}\right)$ of the wheel in practice. Therefore, ${\ddot{z}}_{w}$ is used to represent the influence of wheel dynamic load change. Based on the dynamic characteristics of the suspension system, it can be seen that the three performance indexes influence and restrict each other.

9 $$\left\{\begin{array}{c}min({\ddot{z}}_{b})\\ min({z}_{b-}{z}_{w})\\ min({\ddot{z}}_{w})\end{array}\right.$$

Further, the “fusion reward function” ${R}_{e}$ driven by knowledge-data fusion, consists of two parts, one is based on the performance indexes of the controllable suspension system, and the weight relationship between the performance indexes is considered. The other one considers the feedback effect of robust fault-tolerant control law *C*.

10 $${R}_{e}={R}_{p}+{R}_{c}$$

where ${R}_{p}$ is the comprehensive reward of suspension system performance index, Eqs. (11)–(15). ${R}_{c}$ is the boundary constraint reward of robust fault-tolerant control law *C*, Eq. (16).

11 $${R}_{p}=-\sum_{j=1}^{3}{\omega }_{j}{R}_{i}$$

12 $${\omega }_{j}={\left(1+\sum_{k=2}^{j}\prod_{i=k}^{j}{r}_{i}\right)}^{-1}$$

13 $${R}_{1}=-{{\ddot{z}}_{b}}^{2}$$

14 $$R_{2} = \left\{ \begin{gathered} 0\quad \quad \quad \quad \quad |z_{{b - }} z_{w} | \le \left( {z_{{b - }} z_{w} } \right)_{{\max }} \hfill \\ - (z_{{b - }} z_{w} )^{2} \quad else \hfill \\ \end{gathered} \right.$$

15 $$R_{3} = \left\{ \begin{gathered} 0\quad \;\;\;~\left| {M_{w} \ddot{z}_{w} + M_{b} \ddot{z}_{b} } \right| \le \left( {M_{b} + M_{w} } \right)g \hfill \\ - 50\;\;\;else \hfill \\ \end{gathered} \right.$$

where ${\omega }_{j}$ is the weight coefficient of each performance index of the suspension system, which is determined based on the order relation method, as shown in Eq. (12), where the rational value *r*_*k*_ is assigned according to expert experience, and the value *r*_*2*_ is 1.4, indicating that *R*_*1*_ is significantly more important than *R*_*2*_. The value of *r*_*3*_ is 1.2 means that *R*_*2*_ is slightly more important than *R*_*3*_. Then the weight coefficients ${\omega }_{1}$, ${\omega }_{2}$ and ${\omega }_{3}$ are 0.4430, 0.3093 and 0.2577 respectively.

Where ${R}_{1}$ is represented by the negative square value of the body vertical acceleration, and the greater the value, the greater the reward, that is, the lower the penalty. ${R}_{2}$ triggers different reward values according to the conditions, when the suspension dynamic travel displacement is within the range of expected dynamic travel displacement, the reward is set to 0. Outside this condition, it is represented by the negative square value of the suspension dynamic travel displacement, and the greater the value, the greater the reward, that is, the lower the penalty. ${R}_{3}$ triggers the setting of different reward values according to the conditions, when the dynamic load of the wheel does not exceed the static load of the wheel, the reward is set to 0. Outside of this condition, the reward is set to − 50, that is, a fixed penalty is given. *R*_*c*_ sets the reward trigger condition according to the constraint boundary of the control law, that is, the robust fault-tolerant control law cannot exceed the feasible region of the control current of the adjustable shock absorber, which is [0.3, 1.6]. When the control law is within the feasible region, the reward is set to 0, outside this condition, the reward is set to − 50, that is, a large fixed value penalty is given to avoid it being outside the feasible region.

16 $$R_{c} = \left\{ \begin{gathered} 0\quad \;\;\;0.3 < ~C_{{dd}} \cdot C_{{kd}} < 1.6 \hfill \\ - 50\;\;\;else \hfill \\ \end{gathered} \right.$$

1) Knowledge control law.

The knowledge control law is modified based on the classical SH (Sky-Hook) control strategy^15^. The on–off SH control law ${C}_{a}\left(I\right)$ is:

17 $${C}_{a}\left(I\right)=\left\{\begin{array}{c}{C}_{min }\;if\;{\dot{z}}_{b}({\dot{z}}_{b}-{\dot{z}}_{w})\le 0\\ {C}_{max}\;if\;{\dot{z}}_{b}({\dot{z}}_{b}-{\dot{z}}_{w})>0\end{array}\right.$$

**Figure 11:**
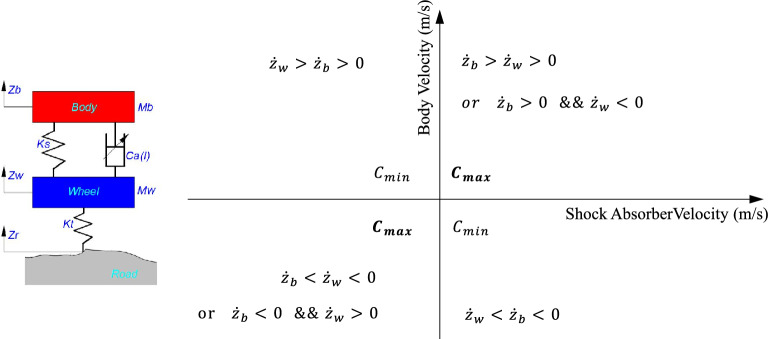
SH control principle.

Figure 11, the body vertical velocity ${\dot{z}}_{b}$ is the vertical coordinate, and the shock absorber velocity ${\dot{z}}_{b}-{\dot{z}}_{w}$ is the horizontal coordinate, which is divided into four quadrants. In the first quadrant, ${\dot{z}}_{b}$ and ${\dot{z}}_{b}-{\dot{z}}_{w}$ are both positive, including two motion cases. One is that the body and the wheel move upward together, and the moving velocity of the body is greater than the wheel. Second, the body moves up but the wheel moves down. In this case, the larger ${C}_{a}\left(I\right)$ is expected from the control requirement, the better, ${C}_{max}$ is taken. In the second quadrant, ${\dot{z}}_{b}$ and ${\dot{z}}_{b}-{\dot{z}}_{w}$ are both positive, the body and wheel move upward together, but the body velocity is smaller than the wheel. In this case, the smaller ${C}_{a}\left(I\right)$ is expected from the control requirement, ${C}_{min}$ is taken. Similarly, the distribution of the third and fourth quadrants is similar.

Based on the analysis of the SH control strategy, it can be concluded that it has a certain conservative passive robust fault tolerance. For example, to ensure adaptability to working conditions, the optimal performance is often sacrificed, so that the control law is according to the current adjustable shock absorber boundary capability ${C}_{max}$ and ${C}_{min}$. When the adjustable shock absorber has perturbation type uncertainty and gain type fault, its damping force value will attenuate. At this time, the control output of the damping force of the shock absorber has reached the adjustable upper limit, and there is no space to increase the control output, to compensate for the corresponding damping force attenuation. Therefore, it can be understood that a certain passive robust fault tolerance has been achieved under the current uncertainty and fault condition.

**Figure 12:**
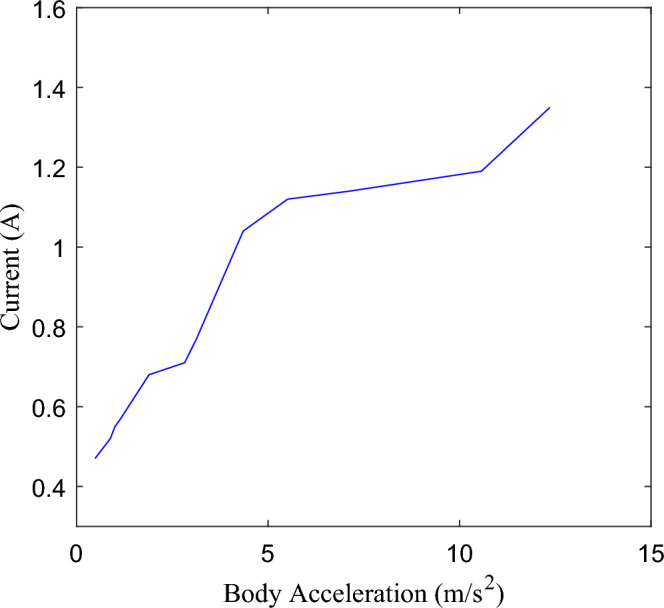
Relationship between *I*_*max*_ and vibration intensity.

Further, combined with Fig. 4b and Eq. (8), ${C}_{max}$ and ${C}_{min}$ are converted into current ${I}_{max}$ and ${I}_{min}$, that is, different currents have different damping coefficient values, and a Map is constructed with the control current and the shock absorber velocity as inputs, and the damping force of the shock absorber as output. If the values of ${I}_{max}$ and ${I}_{min}$ are both constant, the damping control process will always switch repeatedly between the two boundary current states, which is similar to the traditional SH control, and the robust fault tolerance is limited. Therefore, on the premise that ${I}_{min}$ is the lower limit of adjustable current, an interpolation mapping relationship between ${I}_{max}$ and ${\dot{z}}_{b}$ is constructed, as shown in Fig. 12, so that ${I}_{max}$ is correlated with the vibration intensity of the body acceleration, which is related to the change of excitation energy, and ${I}_{max}$ increases monotonically with the vibration intensity. And update the ISH control law, Eq. (18). In this way, it can fully use the adjustable damping force range of the shock absorber, realize the multi-state continuous adjustment of the damping force, improve the performance of the system when there is no fault, and also preset the adjustable space for robust fault-tolerant control.

18 $$C_{a} \left( I \right) = \left\{ {\begin{array}{*{20}c} {I_{{\min ~}} ~~~~~~~~~~~~~if~~~\dot{z}_{b} \left( {\dot{z}_{b} - \dot{z}_{w} } \right) \le 0} \\ {I_{{\max }} \left( {\ddot{z}_{b} } \right)~~~~~\;if~~~\dot{z}_{b} \left( {\dot{z}_{b} - \dot{z}_{w} } \right) > 0} \\ \end{array} } \right.$$

2) Data control law.

Reinforcement learning is a learning method of machine learning, which is a self-learning decision-making method that imitates the learning behavior of animals (including humans). Research has shown that animals (including humans) learn by constantly exploring trial and error, repeating behaviors that bring rewards as much as possible, and avoiding punitive behaviors as much as possible. With the same mindset, reinforcement learning takes action based on feedback from the environment. Through continuous interaction and trial and error with the environment, rewards or punishments are given based on quantitative feedback, ultimately achieving specific goals or maximizing the overall benefits of action. Reinforcement learning means learning “what can be done to maximize the numerical benefit signal^30^.” Based on reinforcement learning does not require labels for training data, can explore unknown fields, and has real-time decision-making ability when interacting with the simulation environment^31,32^, choosing it as the data-driven approach. And select the Proximal Policy Optimization (PPO) algorithm, PPO is a model-free, online, strategy gradient reinforcement learning method that alternates between interactively sampling data through a simulation environment and optimizing tailored proxy objective functions using stochastic gradient descent. The tailored proxy objective function improves training stability by limiting the size of the policy change at each step. The action space can be discrete or continuous, the action space of this subject is continuous. It is based on the actor-critic framework, which is a framework based on the action-value function. Actor learns strategy function *π* and Critic learns action value function *V*. The algorithm steps are shown in Table 3.

**Table 3:** PPO algorithm steps.

1. Initialize the actor $\pi (A|S,\theta )$ with random parameter values *θ*
2. Initialize the critic $V(S,\varnothing )$ with random parameter values *ϕ*
3. Generate ***N*** experiences by following the current policy. The experience sequence is: ${S}_{ts},{A}_{ts},{R}_{ts+1},{S}_{ts+1},\cdots ,{S}_{ts+N-1},{R}_{ts+N-1},{S}_{ts+N},{R}_{ts+N}$
4. For each episode step $t={t}_{s+1},{t}_{s+2},\cdots ,{t}_{s+N}$, compute the return and advantage function Compute the advantage function *D*_*t*_, which is the discounted sum of temporal difference errors: ${D}_{t}=\sum_{k=t}^{{t}_{s+N-1}}{\left(\gamma \lambda \right)}^{k-t}({R}_{t}+b\gamma V({S}_{t},\varnothing ))$ Compute the return *G*_*t*_: ${G}_{t}={D}_{t}+V({S}_{t},\varnothing )$
5. Learn from mini-batches of experiences over ***K*** epochs
a. Sample a random mini-batch data set of size ***M*** from the current set of experiences
b. Update the critic parameters by minimizing the loss ***L***_***critic***_ across all sampled mini-batch data ${L}_{critic}\left(\varnothing \right)=\frac{1}{M}\sum_{i=1}^{M}{({G}_{i}-V({S}_{i},\varnothing ))}^{2}$
c. Normalize the advantage values ***D***_***i***_ based on recent unnormalized advantage values $\widehat{{D}_{i}}\leftarrow \frac{{D}_{i}-mean({D}_{1},{D}_{2},\cdots ,{D}_{M})}{std({D}_{1},{D}_{2},\cdots ,{D}_{M})}$
d. Update the actor parameters by minimizing the actor loss function ***L***_***actor***_ across all sampled mini-batch data ${L}_{actor}\left(\theta \right)=\frac{1}{M}\sum_{i=1}^{M}(-{\text{min}}\left({r}_{i}\left(\theta \right)\cdot {D}_{i},{c}_{i}\left(\theta \right)\cdot {D}_{i}\right))$ ${r}_{i}\left(\theta \right)=\frac{\pi ({A}_{i}|{S}_{i},\theta )}{\pi ({A}_{i}|{S}_{i},{\theta }_{old})}$ ${c}_{i}\left(\theta \right)={\text{max}}({\text{min}}\left({r}_{i}\left(\theta \right),1+\varepsilon \right),1-\varepsilon )$
6. Repeat steps 3 through 5 until the training episode reaches a terminal state

## Demonstrative example

**Figure 13:**
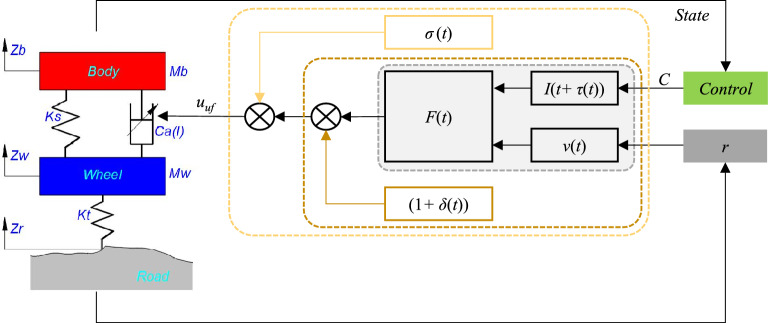
Nonlinear non-stationary uncertain interactive simulation model of the controllable suspension system.

Based on the knowledge-data fusion driven robust fault-tolerant control architecture and strategy proposed in Section “Robust fault-tolerant control architecture and strategy”, and combined with Eqs. (1), (2), (3), and (8), an interactive simulation model can be constructed taking into account the nonlinear, non-stationary, and uncertainty of the controllable suspension system, as shown in Fig. 13. The “*control*” in the figure corresponds to Fig. 10. And set the initial parameters of the training interactive simulation environment as shown in Table 4.

**Table 4:** Initial parameters of the interactive simulation environment.

No.	Items	Remarks
1	Uncertainty—time delay type	$\tau =10 \; {\text{ms}}$
2	Uncertainty—perturbation type	$\left|\delta \right|\le 0.15$
3	Uncertainty—external input excitation	${V}_{el}=30 \; {\text{km}}/{\text{h}}$, speed bump(0-2 s) + B class pavement(2-5 s)
4	Fault—gain type	$\sigma =0.70$
5	Simulation duration	${T}_{f}=5 \; {\text{s}}$
6	Simulation step size	${T}_{s}=0.01 \; {\text{s}}$

**Figure 14:**
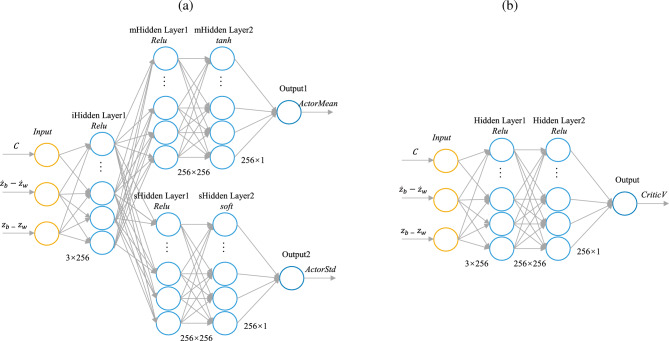
The network: (**a**) *Actor* network, and (**b**) *Critic* network.

The key parameters of the PPO algorithm are set as follows: the discount factor $\gamma$ is 0.95, the learning rate $\alpha$ is 0.001. Actor-Critic network architecture is shown in Fig. 14. The input of *Actor* network is the *Observation* in the previous section. The number of hidden layers is 3, and the number of nodes is 256. The output is defined as the mean and standard deviation of continuous Gaussian probabilities *ActorMean* and *ActorStd*. The activation function of layer 1 and layer 2 is *Relu*, the activation function of Layer 3 corresponding to *ActorMean* is *tanh*, and the activation function of layer 3 corresponding to *ActorStd* is *soft*. The input of *Critic* network is also the *Observation* in the previous section. The number of hidden layers is 2, and the number of nodes is 256. Output is evaluation *CriticV*. All activation functions are *Relu*.

### Training result analysis

**Figure 15:**
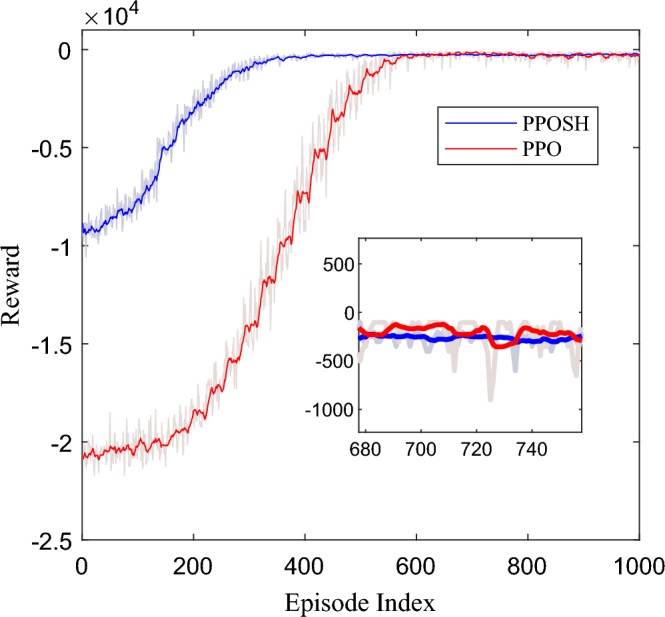
Training iterative process.

Reinforcement learning training is conducted based on the above Settings, and the training iteration process is shown in Fig. 15. The total Episode of iteration is set to 1000, and convergence occurs after about 340 rounds of PPOSH control (knowledge—data driven) iteration, and 600 rounds of PPO control (data driven) iteration. It can be seen that the knowledge-data fusion driven control method can improve the learning convergence speed and shorten the training time.

**Figure 16:**
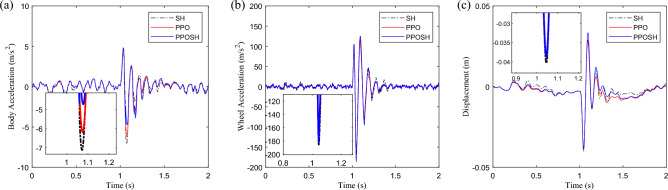
Comparison of time domain data in pulse road excitation section: (**a**) body acceleration, (**b**) wheel acceleration, and (**c**) suspension dynamic displacement.

**Figure 17:**
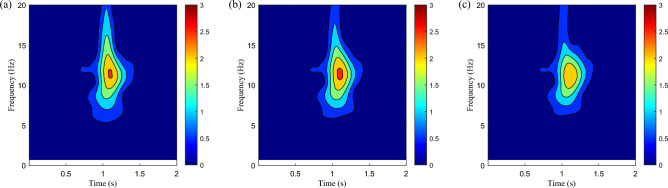
Time–frequency domain data of body acceleration in pulse road excitation section: (**a**) SH control, (**b**) PPO control, and (**c**) PPOSH control.

**Figure 18:**
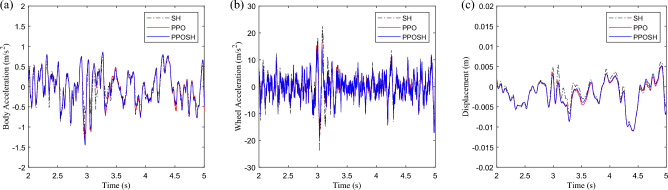
Comparison of time domain data in B-class road excitation section: (**a**) body acceleration, (**b**) wheel acceleration, and (**c**) suspension dynamic displacement.

**Figure 19:**
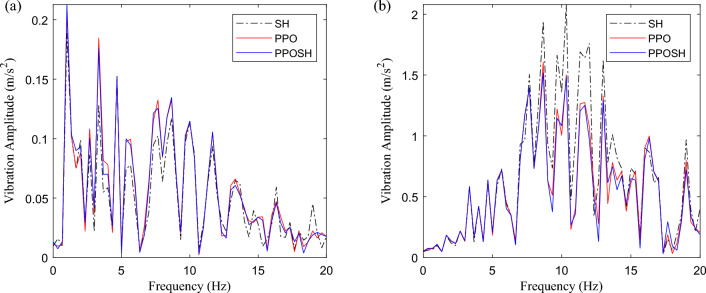
Comparison of frequency domain data in B-class pavement excitation section: (**a**) body acceleration, and (**b**) wheel acceleration.

After the training, the PPOSH control is compared with the SH control (knowledge driven) and the PPO control(data driven). Considering that the defined non-stationary excitation condition is constructed with the B-class road surface and speed bump composite time-domain model, it includes pulse excitation and random excitation. Due to the different processing methods of pulse excitation and random excitation data, the simulation data is divided into two sections according to the definition of road excitation. 0–2 s is mainly defined as the pulse road excitation segment, which is dominated by the speed bump excitation. 2–5 s is mainly B-class road excitation, which is defined as the random B-class road excitation segment. Figure 16 shows the time-domain data results of the pulse road excitation section, in which the excitation energy is large, the response value of vehicle body acceleration is also large, and it presents a transient mutation characteristic. In this case, emphasis is placed on strengthening vibration transmission attenuation, that is targeted reduction of vehicle body acceleration. The results show that the acceleration of the vehicle body is improved obviously when the wheel acceleration and suspension dynamic travel displacement are not significantly different. Figure 17 shows the results of wavelet transform time–frequency analysis of vehicle body acceleration. It can be intuitively seen that in the speed bump excitation time history, the vibration energy distribution is ordered as SH control > PPO control > PPOSH control in the range of 0–20 Hz, and the smaller the vibration energy is, the better the vibration suppression effect is. Figure 18 shows the time-domain data results of the random road excitation section. The excitation energy of this section is small, and the response value of vehicle body acceleration is also small. In this case, the emphasis is placed on strengthening the grounding control of the wheel, and the wheel acceleration can be reduced effectively. Figure 19 shows the Fourier transform frequency domain analysis results of vehicle body acceleration and wheel acceleration. It can be intuitively seen that wheel vibration amplitude is significantly suppressed in the range of 0–20 Hz in the comfort concern band, during the random B-class road excitation period.

**Figure 20:**
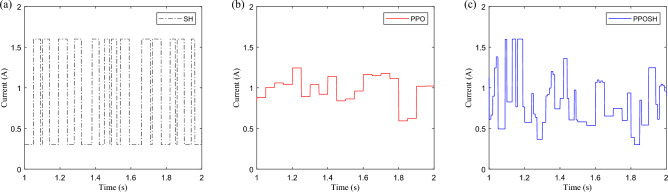
The control law: (**a**) SH control, (**b**) PPO control, and (**c**) PPOSH control.

**Figure 21:**
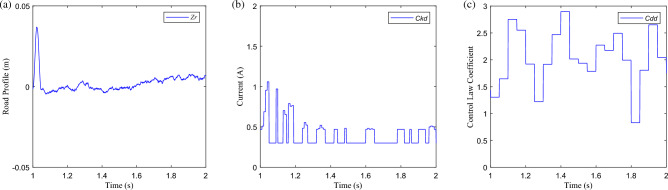
The road excitation and control law: (**a**) road excitation *z*_*r*_, (**b**) knowledge control law *Ckd*, and (**c**) data control law *Cdd*.

Compare the control law of SH, PPO, and PPOSH, and take the data of 1–2 s in the pulse road excitation segment as an example, as shown in Figs. 20 and 21. As shown in Fig. 20a, the SH control law switches between the defined minimum current of 0.3A and the maximum current of 1.6A, according to the rules. This mode is a conservative control mode. Regardless of whether the adjustable shock absorber has fault attenuation of damping force, the output is according to the upper limit capacity of the shock absorber, and has certain conservative robust fault tolerance. As shown in Fig. 20b, the PPO control law switches on demand within the range of adjustable drive current and has a certain adaptive adjustment ability, but it is not strongly related to the excitation condition. As shown in Fig. 21a, the road excitation during the 1–2 s period mainly enters the pulse excitation from 1 s and enters the B-class random road excitation after a short impact. There is no obvious difference between the upper and lower limit of the control law transformation in the pulse excitation segment and the random excitation segment.

But as shown in Fig. 20c, the control law of PPOSH can be adjusted according to the intensity of excitation energy in the range of adjustable current, and it has obvious following in the control law of pulse excitation segment and random excitation segment. It is attributed to the *Cdd*and *Ckd* of PPOSH control law, as shown in Figs. 21b,c, wherein *Ckd* is a knowledge control law defined by Eq. (18), which has strong theoretical support. *Cdd* is a data control law learned based on the PPO strategy, which can obtain proportional compensation for uncertainty and fault in a fuzzy way.

Concretely, Fig. 20c is obtained by multiplying the values of (b) and (c) in Fig. 21, where the knowledge control law *Ckd* is based on the dynamic characteristics of the suspension system and has strong interpretability. With the help of data-driven fuzzy mapping capability, data control law *Cdd* can directly establish a direct correlation between input signal containing uncertainty and control output, obtain proportional compensation for the impact of uncertainty and fault, avoid the process of diagnosis and identification of uncertainty and fault, realize no-estimator control, and simplify the control loop.

**Figure 22:**
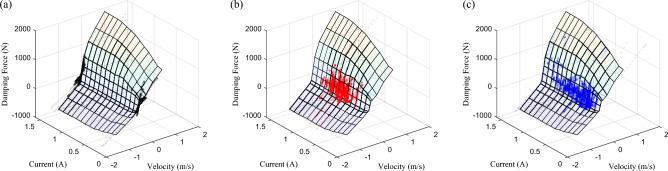
Adjustable damping force distribution: (**a**) SH control, (**b**) PPO control, and (**c**) PPOSH control.

Compare the adjustable damping force distribution of SH, PPO, and PPOSH, and take the data of 0–5 s as an example, as shown in Fig. 22. The order of the utilization rate of each control strategy for the adjustable damping force is PPOSH control > PPO control > SH control. The higher the utilization rate of the damping force is, the better the adaptability of the control strategy in the adjustable range of the damping force.

### Robust fault-tolerant control effect of the suspension system

To evaluate the robust fault tolerance of the proposed knowledge-data-driven control strategy, that is the comprehensive control effect evaluation under various combinations of uncertainty and fault. Uncertainty and fault are defined in Table 5 and combined, *T*_*f*_ = 5 s, *T*_*s*_ = 0.01s. The statistics of each quantitative evaluation index are shown in Tables 6 and 7. Table 6 shows the data statistics of the impulse road excitation input segment. In the case of large and non-stationary road excitation energy, the time-domain peak values of vehicle acceleration under different combinations of uncertainty and fault all show that PPO control and PPOSH control are better than SH control, with an increase of more than 12.97%, and the wheel acceleration in time-domain and dynamic travel displacement in the time-domain increase or decrease very little, within 4.22%. Compared with PPO control, PPOSH control shows obvious advantages in 3 of the 4 combinations, and only the BD combination shows similar advantages. Table 7 shows the data statistics of the random B-class road excitation input segment. When the road excitation energy is small and approximately stable, PPO control and PPOSH control pay more attention to maintaining a clear road sense than SH control. The wheel acceleration time domain root mean square value of PPO control and PPOSH combined with different uncertainty and fault are better than SH control, with an increase of more than 15.37%. Due to the mutual restrictive relationship between vehicle acceleration and wheel acceleration index, the RMS value of vehicle acceleration and dynamic travel displacement in the time domain is increased, and the increase is within 9.43%. Compared with SH control, it can balance the control of body acceleration and wheel acceleration when the value of body acceleration is not large (about 0.3000 m/s^2^). The PPOSH control optimizes wheel acceleration by the same amount as the PPO control in all four combinations.

**Table 5:** Uncertainty and fault definition.

No.	Items	Symbol	Remarks
1	Uncertainty—time delay type	A	$\tau =10 \; {\text{ms}}$
		B	$\tau =20 \; {\text{ms}}$
2	Uncertainty—perturbation type	–	$\left|\delta \right|\le 0.15$
3	Uncertainty—external input excitation	–	${V}_{el}=30 \; {\text{km}}/{\text{h}}$, B-class pavement (random excitation)
		–	${V}_{el}=30 \; {\text{km}}/{\text{h}}$, speed bump (pulse excitation)
4	Fault—gain type	C	$\sigma =0.70$
		D	$\sigma \ge 0.50$

**Table 6:** Pulse road excitation input segment (0-2s).

Performance index	Control method	Combination of uncertainty and fault			
		*G*_*1*_ = AC	*G*_*2*_ = BC	*G*_*3*_ = AD	*G*_*4*_ = BD
*P*_*1*_ (m/s^2^)	SH	6.5993	7.0413	7.1180	7.7272
	PPO	5.3738 (↓18.57%)	5.8698 (↓16.64%)	6.1949 (↓12.97%)	6.3422 (↓17.92%)
	PPOSH	4.6724 (↓29.20%)	4.9781 (↓29.30%)	4.8222 (↓32.25%)	6.5264 (↓15.54%)
*P*_*2*_ (m)	SH	0.0409	0.0394	0.0403	0.0394
	PPO	0.0394 (↓3.67%)	0.0395 (↑0.25%)	0.0387 (↓3.97%)	0.0387 (↓1.78%)
	PPOSH	0.0394 (↓3.67%)	0.0395 (↑0.25%)	0.0386 (↓4.22%)	0.0388 (↓1.52%)
*P*_*3*_ m/s^2^	SH	188.9313	185.9367	186.7408	186.5825
	PPO	184.3249 (↓2.44%)	184.6072 (↓0.72%)	181.3818 (↓2.87%)	181.2455 (↓2.86%)
	PPOSH^2^	184.2881 (↓2.46%)	184.2898 (↓0.89%)	181.3790 (↓2.87%)	181.4809 (↓2.73%)

*In the table, *P*_*1*_ is the acceleration of the body, *P*_*2*_ is the dynamic travel displacment of the suspension, and *P*_*3*_ is the acceleration of the wheel. The parameters in Table 7 are defined in the same way.

**Table 7:** Random B-class road excitation input segment (2-5s).

Performance index	Control method	Combination of uncertainty and fault			
		*G*_*1*_ = AC	*G*_*2*_ = BC	*G*_*3*_ = AD	*G*_*4*_ = BD
*P*_*1*_ (m/s^2^)	SH	0.3098	0.3234	0.2908	0.3011
	PPO	0.3390 (↑9.43%)	0.3406 (↑5.32%)	0.3156 (↑8.53%)	0.3164 (↑5.08%)
	PPOSH	0.3359 (↑8.42%)	0.3389 (↑4.79%)	0.3124 (↑7.43%)	0.3124 (↑3.75%)
*P*_*2*_ (m)	SH	0.0033	0.0038	0.0036	0.0041
	PPO	0.0035 (↑6.06%)	0.0035 (↓7.89%)	0.0038 (↑5.56%)	0.0037 (↓9.76%)
	PPOSH	0.0035 (↑6.06%)	0.0035 (↓7.89%)	0.0038 (↑5.56%)	0.0037 (↓9.76%)
*P*_*3*_ (m/s^2^)	SH	4.8683	5.3112	5.2472	5.5527
	PPO	4.0323 (↓17.17%)	4.0418 (↓23.90%)	4.4406 (↓15.37%)	4.3121 (↓22.34%)
	PPOSH	4.0713 (↓16.37%)	4.0929 (↓22.94%)	4.3564 (↓16.98%)	4.3592 (↓21.49%)

**Figure 23:**
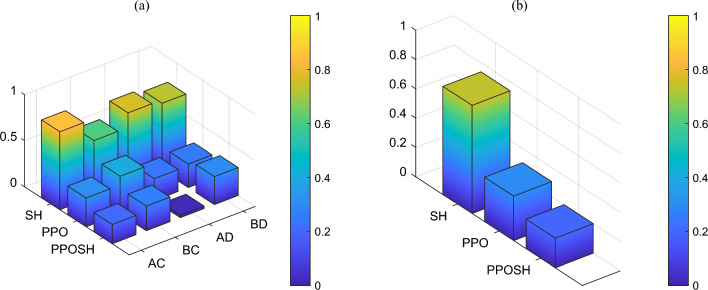
Comparison of index in pulse excitation section: (**a**) the normalized index, and (**b**) the comprehensive index.

**Figure 24:**
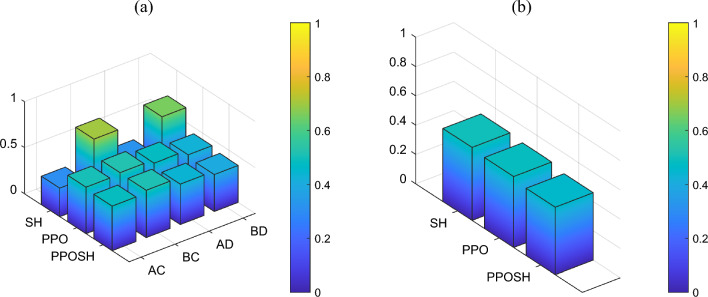
Comparison of index in random B-class excitation section: (**a**) the normalized index, and (**b**) the comprehensive index.

Further, the performance index in Tables 6 and 7 are normalized to obtain the relative value of the three performance indexes (body acceleration, wheel acceleration, and suspension dynamic travel displacement) of SH control, PPO control, and PPOSH control under the combinations of uncertainty and fault, the lower the value, the better the control effect. And the weight coefficient ${\omega }_{i}$ determined by reference to Eq. (15), and the three performance indexes are weighted to obtain the normalized index *P*, Eq. (19). As shown in Figs. 23a and 24a, SH control is superior to PPO control and PPOSH control in the B-class road excitation section (AC and AD combinations), but it deteriorates significantly in BC and BD combinations. Furthermore, considering that the normalized index of a single combination of uncertainty and fault cannot be used as the evaluation index, the four designed combinations of uncertainty and fault are assumed to have equal probability of occurrence, and the mean value of *P* is used as the comprehensive index $\overline{P }$. The results are shown in Table 8, Figs. 23b and 24b. Under the two classes of road excitation, the control effect is PPOSH control > PPO control > SH control, which has a slight advantage under the near stationary excitation, with a maximum increase of 7.43%. Under the non-stationary excitation, the advantageous effect is obvious, and the maximum increase is 72.66%. Therefore, in summary, the proposed knowledge-data driven (PPOSH control) has better fault-tolerant for non-stationary excitation and near-stationary excitation.

19 $$P=\sum_{i=1}^{3}{\omega }_{i}{\cdot nor(P}_{i})$$

where *nor*( ) is a normalized function, *P*_*i*_ is each performance index, *i* = 1, 2, 3, the value is the time domain peak value in the 0–2 s of pulse road excitation segment, and the value is the root mean square value in the 2–5 s of B-class road excitation segment.

20 $$\overline{P }=\frac{1}{N}\sum_{j=1}^{4}{P}_{{G}_{j}}$$

where ${P}_{{G}_{j}}$ is a normalized index under the combination of uncertainty and fault, *j* = 1, 2, 3, 4, *N* = 4.

**Table 8:** Statistical table of the comprehensive index.

	Performance index	SH	PPO	PPOSH
$\overline{P }$	Pulse excitation segment	0.7378	0.3035 (↓58.86%)	0.2017 (↓72.66%)
	Random B-class excitation segment	0.4977	0.4851 (↓2.53%)	0.4607 (↓7.43%)

### Real-time experiment

**Figure 25:**
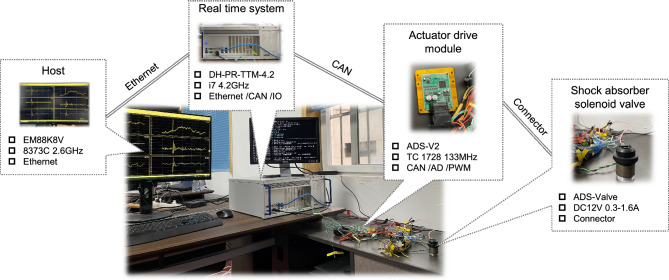
Real-time experiment environment.

**Figure 26:**
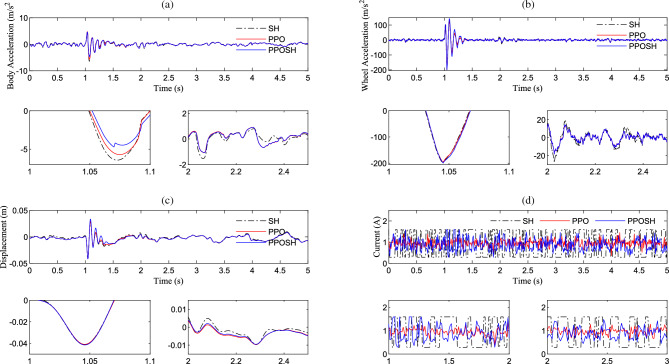
Comparison of time domain data in pulse road excitation section and B-class road excitation section: (**a**) body acceleration, (**b**) wheel acceleration, (**c**) suspension dynamic displacement, and (**d**) control law.

From the perspective of the demand for control methods in actual vehicle applications, the effectiveness and feasibility of a set of control methods are mainly reflected in two aspects: firstly, the control methods can bring performance improvement; secondly, the control method can be deployed for real-time system operation. In this section, an experiment environment will be built based on real-time systems and actuator driver modules to further verify the feasibility of proposing control methods. As shown in Fig. 25, the real-time testing environment consists of a Host (H), Real-time system (RTS), Actor drive module (ADM), and Shock absorber solenoid valve (SASV). H communicates with RTS through ethernet, RTS is connected to ADM through CAN, and ADM and SASV are directly connected through connectors. H serves as the terminal for policy compilation, download, and data export. The compiled control strategy runs in RTS, and the system control load is implemented through ADM and SASV. The type, main parameters, and interface forms of each part are annotated in Fig. 25. Based on this real-time experiment environment, conduct testing and analysis of SH, PPO and PPOSH control. The relevant time-domain data is shown in Fig. 26, in the pulse road excitation section (near the impact peak 1.0–1.1 s), the acceleration of the vehicle body is improved obviously when the wheel acceleration and suspension dynamic travel displacement are not significantly different; in the random road excitation section (taking 2.0–2.5 s as an example), the response value of vehicle body acceleration is low duo to the small excitation energy, the emphasis is placed on strengthening the grounding control of the wheel, and the wheel acceleration can be reduced effectively. Which is consistent with the simulation conclusions described in section “Training result analysis” and “Robust fault-tolerant control effect of the suspension system”. At the same time, the code generation setps including reinforcement learning by MATLAB are shown in Table 9. After the code generation, we can obtain the lines of code for each control method is 578 (SH), 42,824 (PPO), and 44,115 (PPOSH), respectively. It can be found that the knowledge data fusion-driven method proposed in this article, due to the introduction of reinforcement learning, leads to a surge in code size compared to classical control methods. However, fortunately, with the development of hardware technology, resources such as real-time systems can meet the demand for a surge in code size, making the application of knowledge data fusion-driven methods possible. This proves the feasibility of generating and deploying reinforcement learning algorithm code, and the real-time experiment results further validate the effectiveness of the proposed control method.

**Table 9:** The code generation setps including reinforcement learning.

1. Prepare the trained *agent.mat* network by reinforcement learning (*RL*)
2. Load the *agent.mat* and generate *evaluatePolicy.m* by *generatePolicyFunction*()
3. Generate C + + code from the *evaluatePolicy.m* policy script file through *codegen*()
4. Integrate the C + + code into *S-funciton* through the *Legacy code tool*
5. Integrate *RL S-function* and *ISH* to obtain PPOSH control, compile and complete code generation

## Conclusion

A knowledge-data driven robust fault-tolerant control strategy for suspension is proposed. Based on the multiplicity characterization of uncertainty and fault in the established adjustable shock absorber damping force expression, the knowledge control law for system performance optimization is integrated with the data control law to deal with uncertainty and fault. Through the analysis of the training results, the proposed method is verified: on the one hand, it follows the knowledge-driven control law, ensures the interpretability of the control strategy, guarantees the trust in the use of the strategy, provides a basis for troubleshooting and optimization when the control is abnormal, and helps to improve the training convergence speed. On the other hand, it applies the fuzzy mapping capability of data-driven, directly establishes the direct correlation between the input signal containing uncertainty and the control output, obtains the proportional compensation of the influence of uncertainty and fault, avoids the process of diagnosis and identification of uncertainty and fault, realizes no-estimator control, and streamlines the control loop. And based on the weight relationship of three key performance indexes of suspension and different uncertainty and fault combinations hypotheses, a weighted normalized comprehensive index is constructed to evaluate the robust fault-tolerant. The robust fault tolerance control effect is verified by comparing with knowledge-driven control and data-driven control under non-stationary excitation and approximate stationary excitation. The results show that the PPOSH control effect is better under both types of road excitation, with a maximum is 7.43% under approximate stationary excitation and 72.66% under non-stationary excitation. Although the effectiveness and feasibility of the proposed method are verified by the simulation and real-time experiment, the following limitations are worth investigating in the future: (1) The model can be extended to the entire vehicle model, considering the synergy between multiple execution units; (2) Based on combining the method proposed in this article with point (1), the concept of multi-agent collaboration is introduced to research the deployment of actual prototype vehicles.

## Data Availability

The datasets generated and/or analyzed during the current study are available from the corresponding author on reasonable request.
